# Different Temperature and Cooling Patterns at the Blunt and Sharp Egg Poles Reflect the Arrangement of Eggs in an Avian Clutch

**DOI:** 10.1371/journal.pone.0117728

**Published:** 2015-02-06

**Authors:** Miroslav E. Šálek, Markéta Zárybnická

**Affiliations:** Department of Ecology, Faculty of Environmental Sciences, Czech University of Life Sciences Prague, Prague, Czech Republic; SUNY College at Oneonta, UNITED STATES

## Abstract

Incubation is an energetically demanding process during which birds apply heat to their eggs to ensure embryonic development. Parent behaviours such as egg turning and exchanging the outer and central eggs in the nest cup affect the amount of heat lost to the environment from individual eggs. Little is known, however, about whether and how egg surface temperature and cooling rates vary among the different areas of an egg and how the arrangement of eggs within the clutch influences heat loss. We performed laboratory (using Japanese quail eggs) and field (with northern lapwing eggs) experiments using infrared imaging to assess the temperature and cooling patterns of heated eggs and clutches. We found that (i) the sharp poles of individual quail eggs warmed to a higher egg surface temperature than did the blunt poles, resulting in faster cooling at the sharp poles compared to the blunt poles; (ii) both quail and lapwing clutches with the sharp poles oriented towards the clutch centre (arranged clutches) maintained higher temperatures over the central part of the clutch than occurred in those clutches where most of the sharp egg poles were oriented towards the exterior (scattered clutches); and (iii) the arranged clutches of both quail and lapwing showed slower cooling rates at both the inner and outer clutch positions than did the respective parts of scattered clutches. Our results demonstrate that egg surface temperature and cooling rates differ between the sharp and blunt poles of the egg and that the orientation of individual eggs within the nest cup can significantly affect cooling of the clutch as a whole. We suggest that birds can arrange their eggs within the nest cup to optimise thermoregulation of the clutch.

## Introduction

Egg incubation in birds is a complex process that involves the maintenance of a steady egg temperature within the nest to ensure embryonic development [1]. The amount of energy transferred to a developing embryo is mediated by the extent of heat flow from the brood patch of an incubating parent to the eggs, which is achieved through contact between the skin and the egg surface. Excessive heat loss from the egg to the environment is an important issue for bird parents striving to limit energy expenditures during the incubation period [2]. In order to maintain a stable nest microclimate and reduce heat loss, birds can construct specific structural nest configurations [3, 4], nest in cavities [5], or line the nest cup with feathers [6]. Several behavioural adaptations aimed at maintaining an optimal incubation temperature have been described. For example, shortening both the length and frequency of incubation breaks, which accelerates embryo development and reduces the incubation period, are among the behavioural patterns birds used to optimise the incubation process [7].

Egg turning (i.e., egg rotation along the long and short axes) is an activity of the parents during incubation necessary for the successful hatching of most avian embryos [8, 9]. It has been suggested that egg turning prevents the embryo from adhering to the inner shell membrane [7]. Recently, Boulton and Cassey [5] have shown that the temperature on the egg surface and the associated cooling rate (i.e., temperature decline within a defined time interval) are related to the position of the eggs in the nest cup. Specifically, when the clutches are temporarily not provisioned by parents during incubation breaks, the outer eggs exposed to the colder surrounding environment (with a steeper energy gradient) are initially colder and cool more quickly than do the eggs sharing the mutually warmed central positions (and thus exposed to a slower gradient). Egg turning accompanied by exchanging the outer and central eggs within the nest seems therefore to balance heat loss among the eggs and probably constitutes a significant behavioural trait of incubating parents and an integral part of correct embryonic development.

Energy gradients driven by Fourier’s Law of conduction are influenced by both the temperature differential between the environments (here the egg–environment interface) and the shape of the contact zone [2, 10]. Consequently, various segments of the egg surface may have different temperatures and cooling rates depending on their curvature. Avian eggs exist in a variety of shapes [11], and the differences in surface temperatures and cooling rates should be stronger in those eggs with more distinctly shaped blunt and sharp poles. This hypothesis also implies that the arrangement of these eggs (i.e., their orientation within the clutch combined with their exterior or interior position in the clutch) might influence the surface eggshell temperatures and cooling rates of the clutch as a whole. However, this phenomenon has not heretofore been assessed in detail.

Investigation of the thermoregulation patterns of eggs in active clutches of wild birds has been very limited under natural conditions [12]. A pioneering study using an infrared camera was carried out on a cavity passerine, the great tit *Parus major* [5]. An infrared camera can accurately detect the temperature of an eggshell’s surface, and periodic measuring also allows one to follow the temperature dynamics of individual eggs over time. We therefore adopted this approach in our study, which focused on bird eggs with distinctly shaped blunt and sharp poles. We selected two ground-nesting species (the Japanese quail *Coturnix japonica* and the northern lapwing *Vanellus vanellus*) whose nests are naturally exposed to a sharp temperature gradient between the nest cup and ambient environment. As the temperature characteristics might differ between eggs with developed embryos and fresh eggs [2], we included both categories into our study (fresh quail eggs and lapwing eggs in later incubation stages). First, we determined whether the temperature of the eggshell surface and the cooling rates vary between different areas of an egg. Second, we ascertained whether the cooling patterns of the clutches (reflecting heat flow) depend upon the spatial arrangement of individual eggs. In particular, we suggest that (i) the temperature of individual eggs (outside the clutch) is higher at the sharp egg poles than the blunt poles because of faster heat flow on the more curved surface; (ii) in accordance with Fourier’s Law of conduction, the cooling rate of individual eggs (outside the clutch) is faster at the sharp poles because of a steeper gradient between the warm eggshell and the environment; (iii) the temperature at the outer clutch positions is lower than at the inner clutch positions while the cooling rate of the outer positions is faster than that for the inner clutch positions because of different temperature gradients between the clutch and its surroundings, a finding which is in line with the results of Boulton and Cassey [5]; (iv) the temperature and cooling rates of the clutch as a whole depend on the spatial arrangement of the eggs. Specifically, clutches with a prevailing external orientation of the sharp poles will cool faster than will clutches with a prevailing external orientation of the blunt poles, a result that is consistent with hypotheses (i) and (ii).

## Methods

### Ethics statement

The work was approved by the Ministry of Education, Youth and Sports of the Czech Republic (permit No. 18894 / 2009–30). Handling of northern lapwing clutches was part of a capturing and ringing procedure performed under the Ringing Centre of the National Museum in Prague (permit No. 1107). Northern lapwing is not protected under the Czech Ministry of the Environment’s directive 395/1992 (which includes a List of Specially Protected Species). A licence to work with Japanese quail eggs was not needed, as these were commercially distributed fresh eggs intended for consumption.

### Laboratory experiments

**Fig 1:**
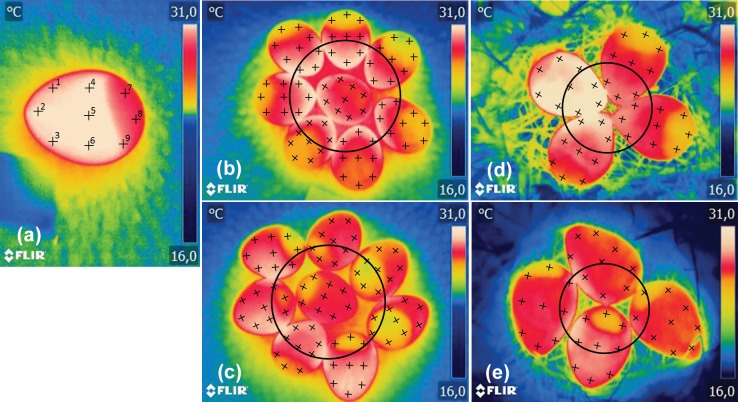
Fixed points (n = 9) located at the sharp pole (points 1 to 3), equator (4 to 6) and blunt pole (7 to 9) of Japanese quail eggs (a). Arranged Japanese quail clutch (i.e., seven eggs with external orientation of blunt poles and one egg in centre of clutch, with fixed points in inner and outer parts of clutch) (b). Scattered Japanese quail clutch (i.e., six eggs with external orientation of sharp poles, one egg with external orientation of blunt pole and one centrally positioned egg, with fixed points in inner and outer parts of clutch) (c). Arranged northern lapwing clutch (i.e., four eggs with external orientation of blunt poles, with fixed points in inner and outer parts of clutch) (d); Scattered northern lapwing clutch (i.e., four eggs with external orientation of sharp poles, with fixed points in inner and outer parts of clutch) (e).

We used fresh Japanese quail eggs that were numbered, measured (length and width; ± 0.05 mm) and weighed (± 0.1 g). The experiments were directed to the individual egg and clutch levels. On the individual level, we recorded the temperature of 17 eggs in isolation from one another. The eggs were placed on a polystyrene foam tray and heated in an incubator to 38°C (the usual incubation temperature in birds; [9]) for 60 min. The heated eggs were individually removed and surface temperatures were immediately recorded using a thermal imager. Two subsequent images were obtained outside the laboratory at an ambient temperature of 12°C after 530.5 ± 9.8 and 1002.3 ± 4.1 s (mean ± SE). The images created with the thermal imager were analysed (see Thermal image processing), and the eggshell surface temperatures at the given times were plotted on a set of nine points located at three areas of each egg: sharp pole, equator and blunt pole (Fig. 1A).

The clutch level measurements utilised a selection of 2 x 8 eggs that were designed to imitate two independent clutches and were placed in a shallow depression within a polystyrene foam tray to simulate the experimental design of Boulton and Cassey [5]. One clutch consisted of seven eggs with an external orientation of the blunt poles and one centrally positioned egg (“arranged clutch”, Fig. 1B), while the second clutch consisted of six eggs with external orientation of the sharp poles, one egg with an externally oriented blunt pole and one centrally positioned egg (“scattered clutch”, Fig. 1C). The egg mass did not differ significantly between the two clutches (Welch test; t = 1.49, DF = 13.94, P = 0.158). Further handling of the clutches was simultaneous and identical. Both of the clutches were placed into an incubator and maintained at 38°C for 60 min. Immediately after heating, the initial temperature was recorded and subsequent measurements were made at repeated intervals of 79.1 ± 3.6 sec (mean ± SE) over a period of 30 min. All of the measurements were made using the thermal imager at an ambient temperature of 12°C. After these measurements were obtained, the clutches were re-arranged into the opposite pattern (scattered to arranged and vice versa) and the experiment was repeated. The images captured with the thermal imager were analysed in the same manner as were the measurements for the individual eggs, with the surface temperature plotted to nine points on each egg. In this case, however, the inner and outer positions of each of the four clutches were defined with a circle (Fig. 1B, C) in order to assign all 72 measurement points to their respective positions in an approximately balanced proportion.

### Field experiment

Two active four-egg northern lapwing clutches found near České Budějovice in the Czech Republic were measured in their later incubation stages (78% and 74% of the incubation period) on 27 April 2014 at 9:00. The egg volumes, which were calculated using the formula 0.457 x L x B^2^, where L is the egg length and B is the egg width [13], differed between the clutches, and the eggs in the first clutch were significantly larger (24.8 ± 0.33 cm^3^ vs. 22.3 ± 0.83 cm^3^, mean ± SE) (Welch test; t = 2.85, DF = 3.90, P = 0.048). The active clutches were substituted with dummy eggs for the time required for measuring, and the clutches were transferred outside the nesting colony of lapwings in a thermobox to avoid excessive disturbance. The eggs were placed in shallow scrapes lined with dry grass in accordance with the real nests. The clutches were covered with a warm blanket and heated at 38°C for 30 min using heat packs. The temperature was continuously monitored using data loggers and manually maintained at a constant value. Subsequently, the temperature was recorded at 71.5 ± 7.8 sec intervals for the following 15 min using a thermal imager. One clutch was positioned as an arranged clutch (all eggs with an external orientation of the blunt pole, Fig. 1D), while the second clutch was positioned as a scattered clutch (external orientation of the sharp poles, Fig. 1E). The arrangement of the clutches was reversed for the second treatment. The images captured using the thermal imager were analysed identically to the measurements for the individual eggs: the surface temperature was plotted to nine points on each egg and, analogous to the quail clutch measurements, the inner and outer positions were defined by a circle (Fig. 1D, E) in order to assign all 36 measurement points to their respective positions in an approximately balanced proportion.

### Thermal image processing

Thermal images were captured using a FLIR E60bx thermocamera with a resolution of 240 x 320 pixels. The camera’s object temperature range is −20°C to 120°C and accuracy is ± 2% of the reading for an ambient temperature between 10°C and 35°C (FLIR System, Inc., Technical data 2013). We set the emissivity to 0.98 [14], photo distance to 0.5 m, and other settings in accordance with the actual temperature and humidity values of the surroundings. After each image was standardised, we imported the image to FLIR Tools version 4.1 user software. We subsequently created fixed spotmeters (the size of a spot used was 6 x 6 pixels) on each egg to display the measurement results (see above).

### Statistical analysis

We applied mixed-effect linear models using the lme4 procedure implemented in the lmer package in R 2.13.1 [15]. The temperature (°C) or cooling rates were included as response variables in the models. Cooling rate was expressed as the slope of the regression line describing the temperature decrease during the time of measurement at a given point of measurement. In the model for temperature patterns in individual quail eggs, we tested the fixed effect of the areas of an egg (sharp pole, equator, blunt pole; defined in Fig. 1) on temperature at nine particular measurement points. The results were controlled for egg mass and time of measurement. The model for cooling rates at particular measurement points in individual quail eggs included the areas of an egg and initial temperature as fixed effects. The model also included the interaction between the areas of an egg and initial temperature, and these results were controlled for egg mass. In the modelling of temperature patterns and cooling rates in quail clutches, we tested the effects of three terms: the fixed effects of egg arrangement (arranged vs. scattered), the positions of measurement points within the clutch (inner vs. outer), and the interaction of these two variables. The time of measurement was controlled in the temperature model, while the initial temperature was controlled in the model for cooling rates. We proceeded to build the models for temperature and cooling rates in lapwing clutches in a manner identical to the procedure used for the quail clutches. The models included the fixed effects of egg arrangement, the position of the measurement point within the clutch, and the interaction of these two variables. The results for the temperature pattern model were controlled for time of measurement, while the cooling rate model was controlled for initial temperature. In the models dealing with individual eggs, the individual egg was considered a random effect while the clutch was considered a random effect in the remaining models dealing with clutches. Tukey’s post hoc multiple comparisons [16] were applied to examine the significance of differences in the temperature and cooling rates of the quail eggs, arranged and scattered clutches, and the inner and outer positions of quail and lapwing clutches.

## Results

### Temperature and cooling rates in Japanese quail eggs

**Fig 2:**
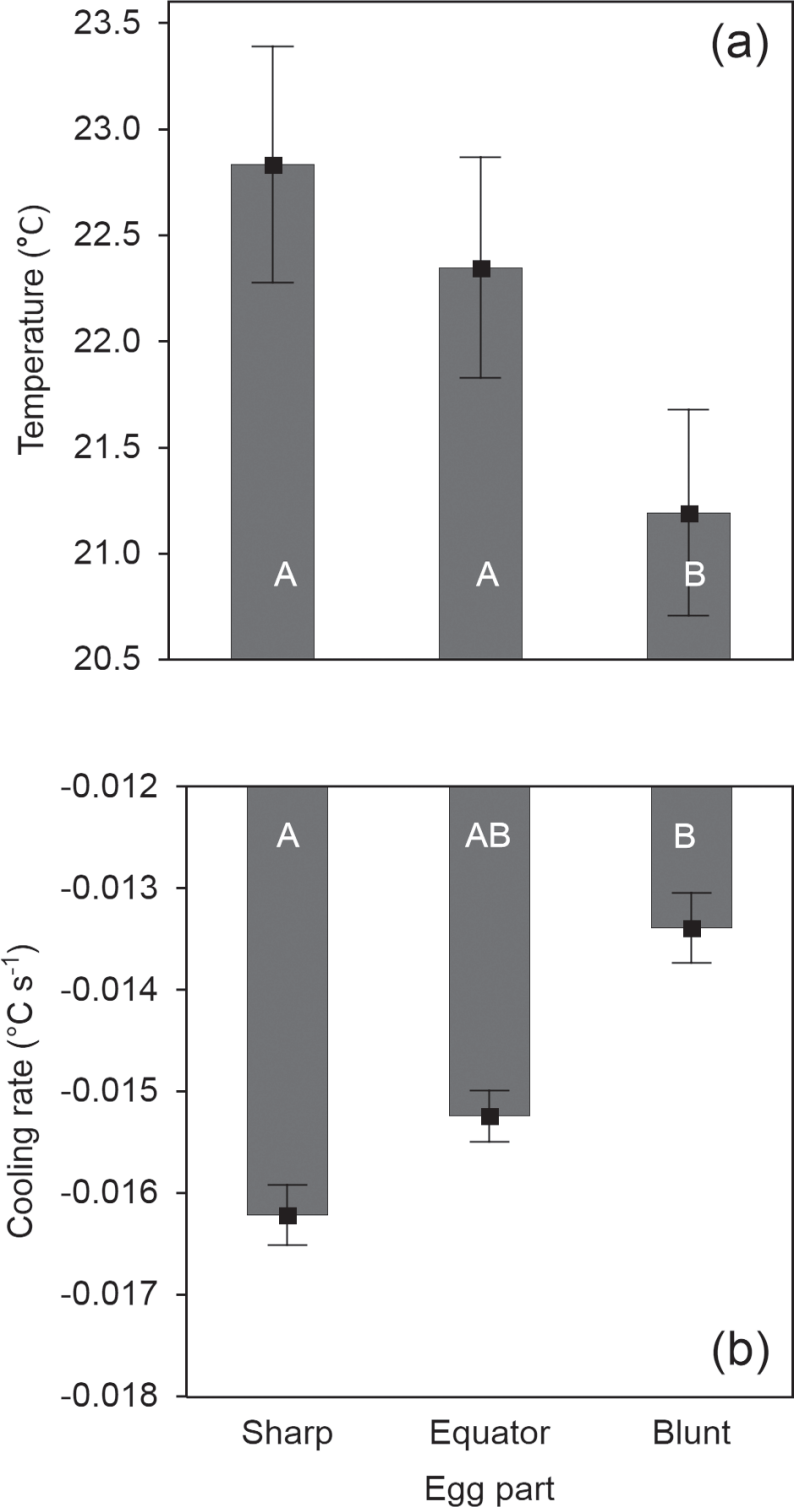
Temperature (a) and cooling rates (b) of three areas (sharp pole, equator, blunt pole) of Japanese quail eggs (n = 17). Means ± SE are given. Eggs were heated to 38°C and subsequently cooled in ambient temperature (12°C). Measurements were recorded at three time intervals (immediately after heating and 530.5 ± 9.8 and 1002.3 ± 4.1 s after heating). Results are controlled for egg mass and time of measurement. Identical and different letters inside the bars, respectively, indicate non-significant and significant differences at P ≤ 0.05 (Tukey’s multiple comparisons).

**Fig 3:**
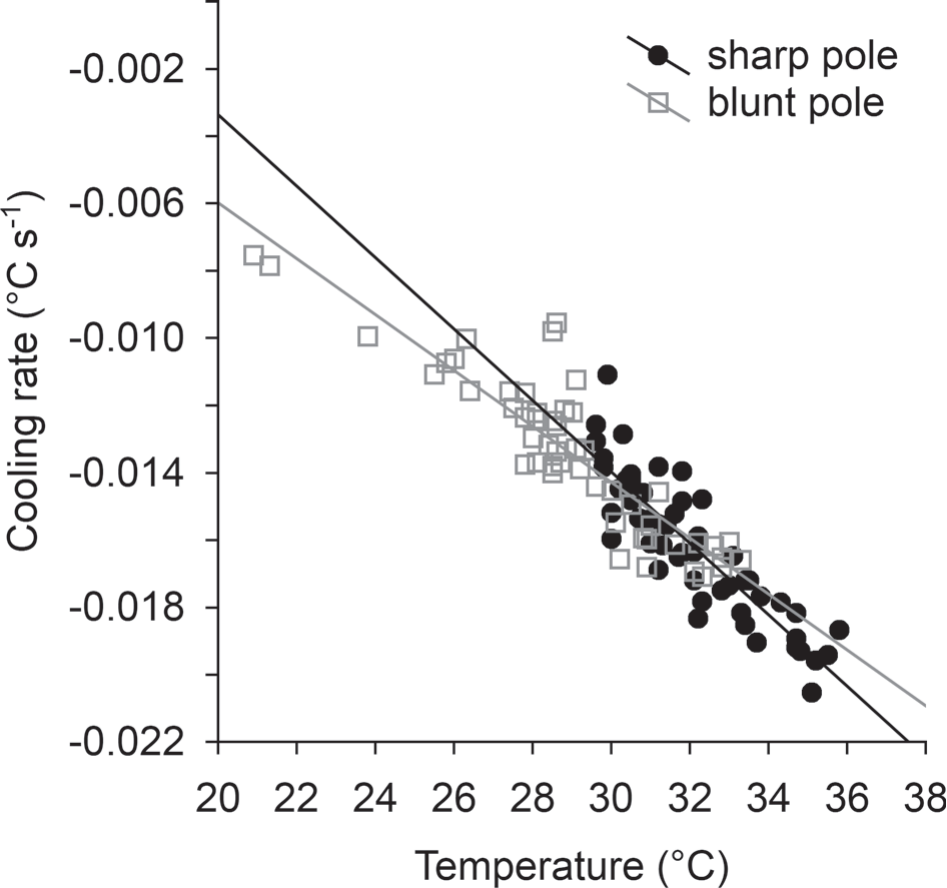
Relationship between egg surface temperature and cooling rate of Japanese quail eggs (sharp and blunt poles).

We found significantly different temperature and cooling patterns in different areas of evenly heated quail eggs (Table 1). Temperature increased from the blunt pole (with the lowest values) to the sharp pole (with the highest values; Fig. 2A). The mean temperatures of the blunt pole and the sharp pole were significantly different (difference between mean temperatures: △t = 1.64°C), as were the temperatures of the blunt pole and the equator (△t = 1.16°C; Table 2, Fig. 2A). The cooling rate (expressed as a regression slope) was strongly affected by the initial temperature, with significantly faster cooling occurring from higher initial temperatures (Table 1, Fig. 3). However, the significant interaction between initial temperature and areas of an egg (Table 1) exhibits different patterns in various areas of the eggs. In general, the blunt pole cooled more slowly than did the sharp pole (Fig. 2B), which effect was particularly obvious at higher initial temperatures (Table 2, Fig. 3).

**Table 1:** Effect of areas of an egg (sharp pole x equator x blunt pole) on temperature and effect of areas of an egg and initial temperature on cooling rates of Japanese quail eggs (n = 17).

Effect on	Factor	Estimate	SE	DF	Χ^2^	P
temperature ^1^	areas of an egg			2, 5	39.34	<0.001
	sharp pole vs. equator	-0.4869	0.2639			
	sharp pole vs. blunt pole	-1.6425	0.2634			
cooling rate ^2^	areas of an egg			2, 5	0.87	0.647
	temperature	-0.0009	0.0001	2, 5	203.71	<0.001
	areas of an egg x temperature			2, 7	6.06	0.048
	sharp pole vs. equator ^3^	0.0002	0.0001			
	sharp pole vs. blunt pole ^3^	0.0001	0.0001			

Eggs were heated to 38°C and subsequently cooled at ambient temperature of 12°C. Measurements were recorded at three time intervals (immediately after heating and 530.5 ± 9.8 and 1002.3 ± 4.1 s after heating).

^1^ Controlled for egg mass and time of measurement

^2^ Controlled for egg mass

^3^ Interaction between areas of an egg and temperature

**Table 2:** Results of Tukey’s tests for multiple comparisons of temperature and cooling rates between different areas of Japanese quail eggs and between internal (int) and outer (out) positions of arranged and scattered Japanese quail and northern lapwing clutches.

Subject		Egg pole / clutch arrangement	Estimate	SE	t-value	P
quail egg	temperature ^1^	blunt vs. sharp pole	-1.6425	0.2639	-6.223	<0.0001
		blunt pole vs. equator	-1.1556	0.2639	-4.378	<0.0001
		equator vs. sharp pole	-0.4869	0.2639	-1.845	0.1550
	cooling rate ^2^	blunt vs. sharp pole	-0.0072	0.0031	-2.357	0.0500
		blunt pole vs. equator	-0.0042	0.0032	-1.315	0.3854
		equator vs. sharp pole	-0.0030	0.0038	-0.771	0.7182
quail clutch	temperature ^3^	arranged int vs. arranged out	2.1665	0.0627	34.55	<0.0001
		scattered int vs. scattered out	0.6595	0.0624	10.563	<0.0001
		arranged int vs. scattered int	-1.2758	0.0672	-18.996	<0.0001
		arranged out vs. scattered out	0.2313	0.0576	4.012	0.0004
	cooling rate ^4^	arranged int vs. arranged out	0.0015	0.0002	9.279	<0.0010
		scattered int vs. scattered out	0.0011	0.0002	6.672	<0.0010
		arranged int vs. scattered int	-0.0009	0.0002	-5.082	<0.0010
		arranged out vs. scattered out	0.0004	0.0001	-2.910	0.0187
lapwing clutch	temperature ^3^	arranged int vs. arranged out	1.9931	0.1386	14.38	<0.0001
		scattered int vs. scattered out	0.7466	0.1423	5.249	<0.0001
		arranged int vs. scattered int	-1.3139	0.1599	-8.218	<0.0001
		arranged out vs. scattered out	-0.0675	0.1249	-0.541	0.9490
	cooling rate ^4^	arranged int vs. arranged out	0.0016	0.0005	3.025	0.0131
		scattered int vs. scattered out	0.0000	0.0005	-0.048	1.0000
		arranged int vs. scattered int	-0.0036	0.0006	-6.008	<0.001
		arranged out vs. scattered out	-0.0019	0.0005	-4.143	<0.001

^1^ Controlled for egg mass and time of measurement

^2^ Controlled for egg mass

^3^ Controlled for time of measurement

^4^ Controlled for initial temperature

### Temperature and cooling rates in Japanese quail clutches

**Fig 4:**
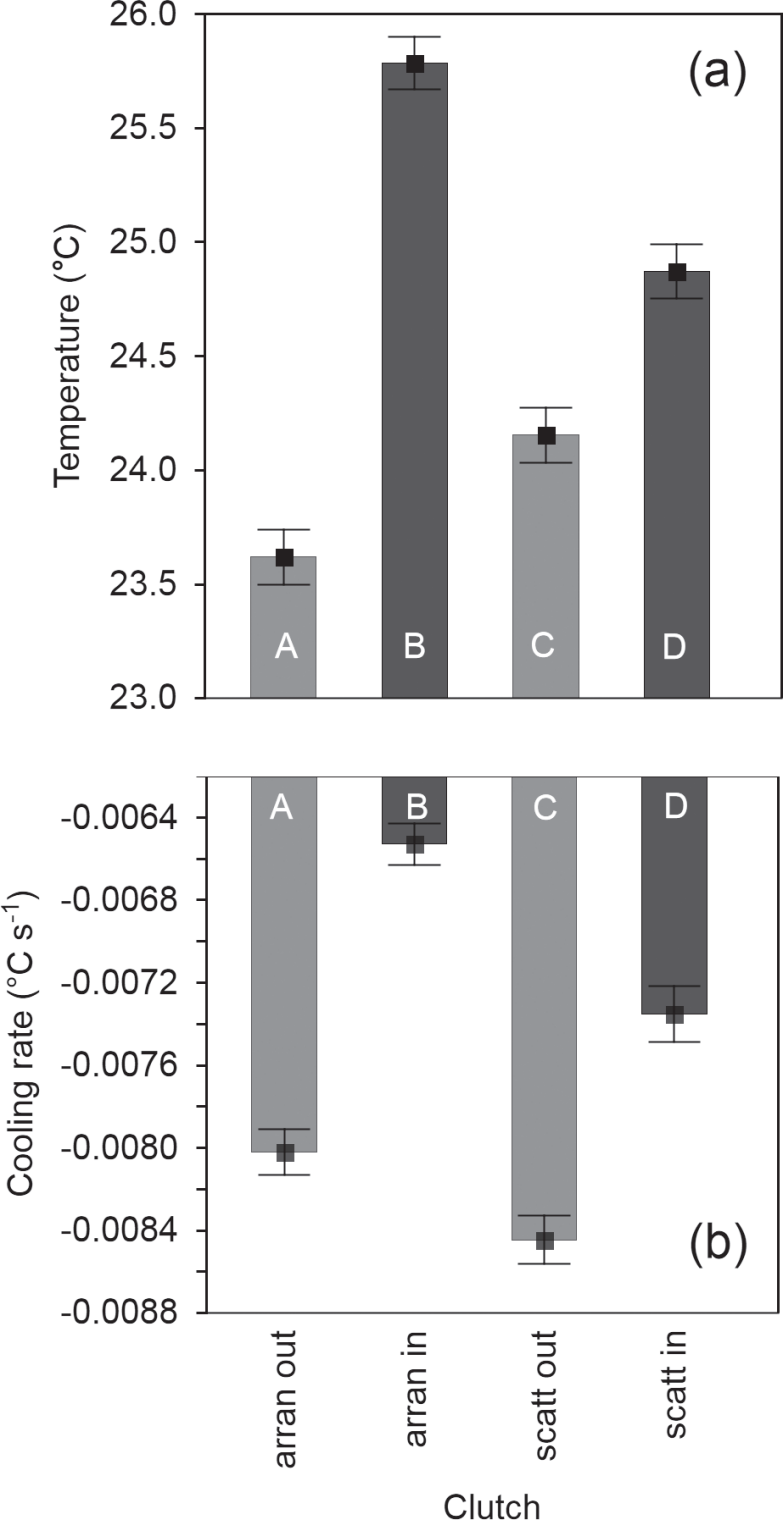
Temperature (a) and cooling rates (b) of arranged (n = 2) and scattered (n = 2) Japanese quail clutches at inner and outer positions. Means ± SE are given. Clutches were heated to 38°C and subsequently cooled in ambient temperature (12°C). Measurements were recorded at 79.1 ± 3.6 s intervals (mean ± SE) over a period of 30 min. Identical and different letters inside the bars, respectively, indicate non-significant and significant differences at P ≤ 0.05 (Tukey’s multiple comparisons).

The arrangement of quail eggs in the nest cup significantly affected the temperature and cooling rates of the inner and outer positions of the clutch, but it did so in different ways (significant interaction, Table 3). In both arranged and scattered clutches, the temperature was significantly higher at inner than at outer positions (Table 2; Fig. 4A), but the difference between the inner and outer positions was greater in the arranged clutches (△t = 2.17°C) than the scattered clutches (△t = 0.72°C). Moreover, the mean temperature was significantly higher at the inner positions of arranged clutches than at the inner positions of scattered clutches (△t = 0.91°C). The outer positions showed the opposite trend, as the outer mean temperature in arranged clutches was significantly lower than the outer mean temperature in scattered clutches (△t = 0.53°C; Table 2, Fig. 4A). The cooling rates of the outer clutch positions were significantly faster than were those at the inner positions in both arranged and scattered clutches (Table 2, Fig. 4B). Furthermore, the cooling rates of the outer and inner positions of the arranged clutches were significantly slower than were those for the respective parts of the scattered clutches (Table 2, Fig. 4B).

**Table 3:** Effects of egg arrangement (arranged or scattered) and clutch position (inner or outer part) on temperature and cooling rates of Japanese quail clutches (n = 2).

Effect on	Factor	Estimate	SE	DF	Χ^2^	P
temperature ^1^	Position	2.1660	0.0627	1, 5	917.370	<0.0001
	Arrangement	0.2313	0.0576	1, 5	83.192	<0.0001
	position x arrangement	-1.5070	0.0885	1, 6	284.650	<0.0001
cooling rate ^2^	position	-0.0004	0.0001	1, 4	95.630	<0.0001
	arrangement	0.0015	0.0002	1, 4	27.269	<0.0001
	position x arrangement	0.0004	0.0002	1, 5	4.2497	0.0393

Clutches were heated to 38°C and subsequently cooled in ambient temperature (12°C). Measurements were recorded at 79.1 ± 3.6 s intervals (mean ± SE) for a period of 30 min.

^1^ Controlled for time of measurement

^2^ Controlled for initial temperature

### Temperature and cooling rates in lapwing clutches

**Fig 5:**
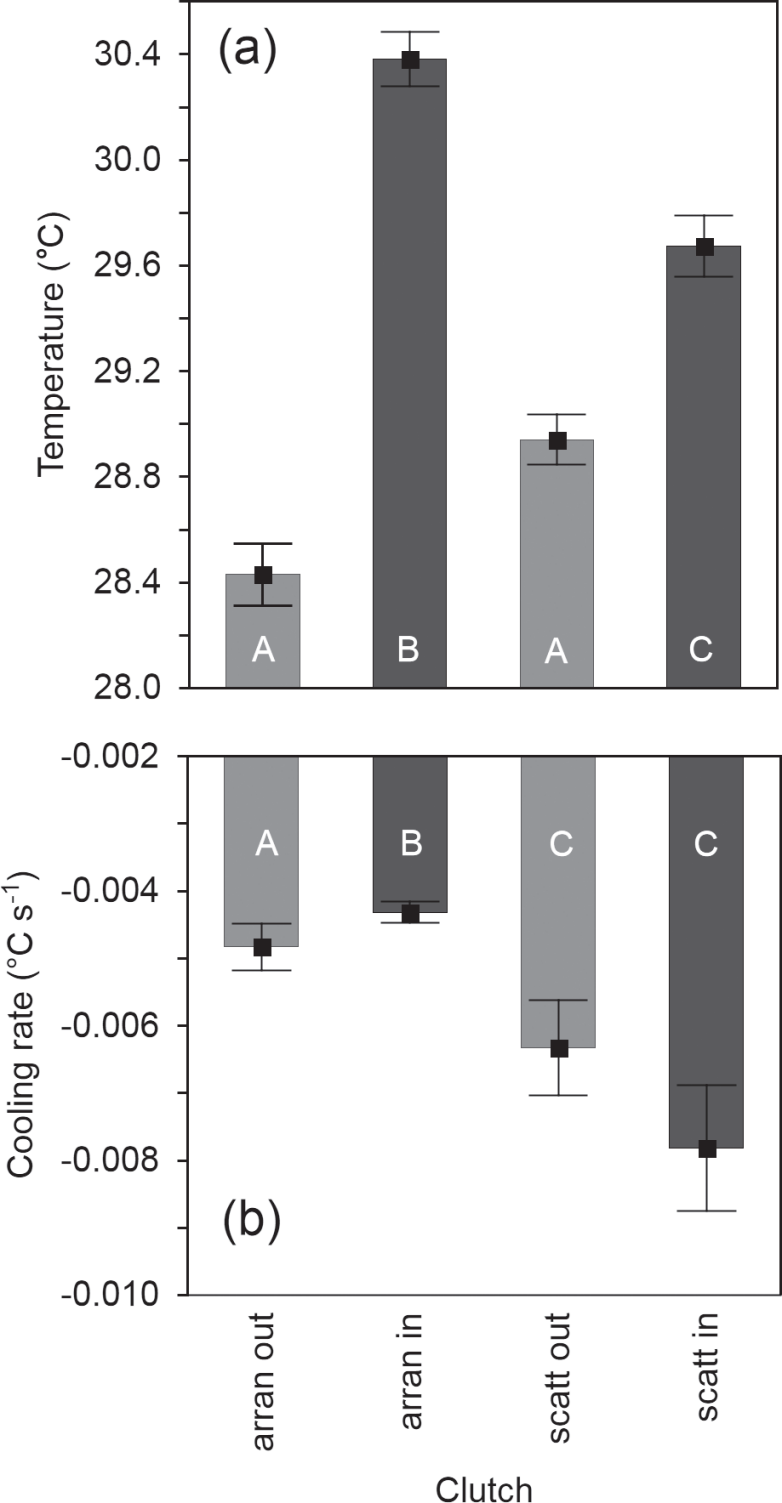
Temperature (a) and cooling rates (b) of arranged (n = 2) and scattered (n = 2) northern lapwing clutches at inner and outer parts of clutches. Means ± SE are given. Clutches were heated to 38°C and subsequently cooled in ambient temperature (20°C). Measurements were recorded at 71.5 ± 7.8 s intervals (mean ± SE) over a period of 15 min. Identical and different letters, respectively, inside the bars indicate non-significant and significant differences at P ≤ 0.05 (Tukey’s multiple comparisons).

Similar to the quail clutches, the arrangement of lapwing eggs in the nest cup affected the temperature and cooling rates at the inner and outer positions of the clutch and exhibited a significant interaction (Table 4). In both the arranged and scattered clutches, the temperature was significantly higher at the inner positions than at the outer positions (Table 2; Fig. 5A), but the difference between the inner and outer positions was greater in the arranged clutches (△t = 1.90°C) than in the scattered clutches (△t = 0.50°C). The significant interaction between position and arrangement indicates a different temperature pattern for the outer and inner positions in the arranged and scattered clutches. Whereas the temperature of the inner positions of the arranged clutches was significantly higher than that of the inner positions of the scattered clutches (△t = 0.77°C), the difference in temperature between the outer positions of the arranged and scattered clutches was not significant (Table 2, Fig. 5A). Furthermore, we found that the outer positions of the arranged clutches cooled faster than did the inner positions (Table 2, Fig. 5B), but no such effect was found in scattered clutches (Table 2). Similar to quail clutches, the arrangement of the lapwing eggs contributed significantly to the cooling pattern, as both the inner and outer positions of the arranged clutches cooled significantly more slowly than did the respective positions in scattered clutches (Table 2, Fig. 5B).

**Table 4:** Effects of egg arrangement (arranged or scattered) and clutch position (inner or outer part) on temperature and cooling rates of northern lapwing clutches (n = 2).

Effect on	Factor	Estimate	SE	DF	Χ^2^	P
temperature ^1^	position	1.9931	0.1386	1, 5	178.160	<0.0001
	arrangement	-0.0675	0.1249	1, 5	27.170	<0.0001
	position x arrangement	-1.2464	0.1986	1, 6	38.970	<0.0001
cooling rate ^2^	position	0.0016	0.0005	1, 5	4.218	0.0400
	arrangement	-0.0019	0.0005	1, 5	41.407	<0.0001
	position x arrangement	-0.0017	0.0008	1, 6	4.909	0.0267

Clutches were heated to 38°C and subsequently cooled in ambient temperature (20°C). Measurements were recorded at 71.5 ± 7.8 s intervals (mean ± SE) for a period of 15 min.

^1^ Controlled for time of measurement

^2^ Controlled for initial temperature

## Discussion

We used infrared imaging to demonstrate different temperature and cooling rate patterns at different areas of experimentally heated quail eggs. We consider these differences to be a consequence of Fourier’s Law of conductance [10]. The more curved, sharp pole with its relatively large surface area per unit egg volume at the tapered portion of the egg probably suffers from greater heat loss during cooling than does the less curved and more bulky blunt pole. The cooling rates then accelerate as the temperature gradient between the egg’s surface and the surrounding environment increases, with more pronounced cooling rates at higher initial incubation temperatures. We suggest that the sharp poles are exposed to a relatively colder surrounding environment and tend to cool more rapidly than do the colder blunt poles exposed to the same but relatively moderate temperature gradient.

Our subsequent experiments with artificially organised quail and lapwing clutches supported the hypotheses as to the effects of egg position (inner and outer parts of the clutch [5]) and egg arrangement (orientation of the eggs relative to the clutch centre; this study) on the temperature and associated cooling patterns within the clutches. Although the experiments with quail clutches were performed in the laboratory and the lapwing trial was conducted in the field, the results consistently indicated that the clutches with the sharp poles oriented towards the clutch centre (arranged clutches) have significantly higher temperatures in the central part of the clutch than do the clutches that have the sharp egg poles turned out (scattered clutches). Consequently, the temperature difference between the inner and outer parts of the clutches was greater in the arranged clutches than in the scattered clutches.

The cooling of quail and lapwing clutches showed a similar pattern. In particular, both the inner and outer parts of the arranged clutches maintained a more stable temperature over time (lower cooling rate) than did the same parts of the scattered clutches. Moreover, the outer clutch positions cooled faster than did the inner positions when the sharp poles were oriented towards the centre of the clutch. The same pattern was found in scattered quail clutches but not in scattered lapwing clutches, where the difference was not significant. The explanation for this slight disparity may be threefold: First, the lapwing experiment was conducted at a higher ambient temperature than was the quail experiment. Therefore, the temperature gradients above the centres and edges of the lapwing clutches were less sharp than were those above the quail clutches. Lending support to this explanation is the fact that we failed to find a significant difference in the surface temperatures at the outer positions of scattered and arranged lapwing clutches. Second, the lapwing eggs were measured at later incubation stages and a more developed embryo could influence the thermal balance of the eggs [5]. Third, the shape of quail and lapwing eggs differ and that could contribute to the differences in egg cooling patterns. It is nevertheless clear that the configuration of the eggs in the nest cup considerably influences the heat loss from the clutches of both species, despite this minor difference in cooling patterns.

In this study, we demonstrated how the surface temperature and cooling rate of eggshells vary among the different areas of eggs and how the organisation of eggs in the nest cup may affect the heat lost by the entire clutch in two precocial ground-nesting species with dissimilar egg shapes. It may be very useful for these species to know how to arrange the eggs inside the nest cup, because the temperature around the nests can vary greatly due to wind, wetness and sunshine. This, in turn, can strongly contribute to the heat balance in the nests of these openly nesting birds [17] in comparison to that of, for example, cavity nesters [18]. Although these findings reflect a basic physical law concerning the effect of surface curvature on heat loss from an object (the egg), it might have far-reaching implications for ecology of breeding birds and for methodologies applied to specific tasks regarding the associated topics.

Regardless of various environmental obstacles that prevent maintaining a constant heat balance in actual bird nests, surprisingly high egg hatchability occurs in the wild [19]. Nevertheless, exposure to temperatures below and above the range of thermal tolerance harms the health and reduces the condition of hatched young while negatively affecting their fitness [19]. On the other hand, repetitive acute cold exposures during the last phase of embryogenesis can improve the ability of growing and cold resistance of experimentally treated broilers after hatching [20]. Therefore, a regular breeding process cannot occur without there being very good thermoregulating mechanisms within the clutch. In addition to the turning of eggs along the long axis and their rearrangement in the clutch to provide for even heating of all eggs [7–9], the turning of eggs with their sharp poles towards the centre or edge of the clutch depending on actual temperature in the nest might be a simple but crucial behavioural trait of nesting birds. To study such behaviour, however, would require experimental manipulation with the eggs while considering variation in the actual surrounding temperature. As it is difficult to study, it is possible that this phenomenon has been overlooked heretofore even though this might have important evolutionary consequences (e.g., as a proxy in the evolution of egg shape combined with clutch size [21], which became surprisingly characteristic in some birds). For example, shorebirds (Limicolae), which have a distinctive clutch of (usually) four very specifically shaped eggs, organise their clutches almost exclusively with the sharp poles centred in the nest cup [22]. We suggest that this symmetric centring reduces the contact area between the sharp poles and the cooler surrounding environment, and thus functions together with other nest attributes as nest lining and cover [17, 23] to improve the heat savings of the entire clutch. Environments with extreme weather conditions such as the high Arctic, where shorebirds commonly breed, may cause adaptations such that eggs have differentiated shapes and parental behaviour corresponds accordingly.

The next step for researchers should therefore be to examine variation in bird incubation behaviour, including parental care for eggs in accordance with the physical law as we have demonstrated. In particular, this should examine which bird species or populations (with regard to geographic range) and at what times (according to incubation phase, weather conditions, and clutch size) (i) arrange their eggs in the clutches according to egg shape, nest characteristics, and actual microclimatic conditions, and (ii) can suffer from potentially detrimental environmental factors which can disrupt the complex of nesting behavioural traits associated with thermoregulation. For example, birds’ chronic exposure to organic pollutants might influence production of thyroid hormone and prolactin with adverse effect on body condition [24, 25] and incubation behaviour [26, 27], inclusive of the ability correctly to manipulate eggs. Another experiment has shown, for example, that glaucous gull (*Larus hyperboreus*) parents are unable to maintain the nest temperature in experimentally enlarged clutches, and this probably is due to change in the physical structure of the clutch [28]. Egg arrangement in the clutch thus should be considered at least as a covariate in models where detailed effects on heat loss are examined.

## Supporting Information

S1 Dataset Primary data used to analyses of temperature and cooling rates of northern lapwing and Japanese quail eggs and clutches. (XLSX)
